# A phase II study evaluating the preventive effect of topical hydrocortisone for capecitabine-induced hand-foot syndrome in patients with colorectal cancer receiving adjuvant chemotherapy with capecitabine plus oxaliplatin (T-CRACC study)

**DOI:** 10.1007/s10147-025-02857-4

**Published:** 2025-08-14

**Authors:** Yohei Iimura, Keisuke Baba, Naoki Furukawa, Masaaki Ishibashi, Chieko Sasuga, Yuka Ahiko, Satoko Monma, Naoki Sakuyama, Susumu Aikou, Dai Shida, Masanori Nojima, Seiichiro Kuroda, Narikazu Boku

**Affiliations:** 1 Department of Pharmacy, The IMSUT Hospital, The Institute of Medical Science, The University of Tokyo, 4-6-1, Shirokanedai, Minato-ku, Tokyo, 108-8639 Japan; 2 Department of Oncology and General Medicine, The Institute of Medical Science Hospital, The University of Tokyo, 4-6-1, Shirokanedai, Minato-ku, Tokyo, 108-8639 Japan; 3 Division of Frontier Surgery, The Institute of Medical Science, The University of Tokyo, 4-6-1, Shirokanedai, Minato-ku, Tokyo, 108-8639 Japan; 4 Department of Surgery, The IMSUT Hospital, The Institute of Medical Science, The University of Tokyo, 4-6-1, Shirokanedai, Minato-ku, Tokyo, 108-8639 Japan; 5 Center for Translational Research, The Institute of Medical Science, The University of Tokyo, 4-6-1, Shirokanedai, Minato-ku, Tokyo, 108-8639 Japan

**Keywords:** Capecitabine-induced hand-foot syndrome, Prevention, Medium-class topical corticosteroids, Colorectal cancer

## Abstract

**Background:**

Topical steroids may help prevent capecitabine-induced hand-foot syndrome in patients with colorectal cancer, as inflammation is involved in anti-tumor agent-induced hand-foot syndrome development. We assessed the preventive efficacy of medium-class topical corticosteroids for capecitabine-induced hand-foot syndrome in patients receiving adjuvant chemotherapy for colorectal cancer.

**Methods:**

This open-label, single-arm, single-center phase II study included patients with colorectal cancer receiving adjuvant chemotherapy with capecitabine + oxaliplatin. Prophylactic topical hydrocortisone butyrate (0.1%) was applied to the palms and soles from day 1 of adjuvant chemotherapy. The primary endpoint was grade ≥ 2 hand-foot syndrome incidence within four cycles. Secondary endpoints were the time to onset and incidence of each hand-foot syndrome grade, dose reduction, schedule delay, hand-foot syndrome-induced discontinuation, and other adverse events.

**Results:**

Fifty patients were enrolled; three were excluded. Among the 47 included (median age = 54.5 years), 100% had Eastern Cooperative Oncology Group performance status 0, 36.1% were male, and 95.7% had pathological stage III disease. Hand-foot syndrome induced dose reduction, schedule delay, and discontinuation were required in 0, 2, and 0 patients, respectively, with a median relative capecitabine dose intensity of 100% within four cycles. Grade ≥ 2 hand-foot syndrome incidence during the cycles was 6.4%. Time to onset of grades ≥ 1 and ≥ 2 was 63.5 and 105.5 days, respectively. One patient experienced grade 3 hand-foot syndrome on day 164. The most common grade ≥ 2 adverse events were peripheral sensory neuropathy and neutropenia. No topical hydrocortisone butyrate (0.1%)-induced adverse events occurred.

**Conclusions:**

Topical hydrocortisone butyrate (0.1%) may prevent capecitabine-induced hand-foot syndrome.

**Trial registration:**

Trial registration number and date of registration: This clinical trial is registered in the Japan Registry of Clinical Trials (jRCT) as jRCTs031220002).

## Introduction

Hand-foot syndrome (HFS) is a clinically relevant adverse effect induced by capecitabine. Randomized controlled trials (RCTs) of capecitabine-containing chemotherapy for colorectal cancer patients revealed incidences of any grade capecitabine-induced HFS ranging from 30 to 80% [1–3], whereas it was reportedly > 90% in the real world [4]. Grade ≥ 2 HFS, characterized by symptoms such as swelling, blistering, skin peeling, and ulcer formation, deteriorates patients’ quality of life [5].

Combination chemotherapy with oral capecitabine plus oxaliplatin (CapeOX) and infusion of 5-fluorouracil, leucovorin, and oxaliplatin (FOLFOX) are two recommended treatment options for adjuvant chemotherapy of pathological stage III colorectal cancer [6]. However, CapeOX is sometimes used with caution in clinical practice due to concerns about HFS. HFS frequently causes treatment interruption, schedule delay, dose reduction, and capecitabine discontinuation. In particular, HFS affects patient adherence to treatment [7], while capecitabine dose intensity is crucial for achieving optimal outcomes in adjuvant chemotherapy for colon cancer [8]. Therefore, prevention and management of HFS are important [9].

Evidence supporting the prevention of capecitabine-induced HFS remains limited. While the preventive efficacy of exfoliating agents has shown inconsistency across clinical trials [10, 11], celecoxib [12, 13] and pyridoxine [14] have been reported to help mitigate HFS. Although celecoxib demonstrated a preventive effect on HFS in a phase III study, its widespread use has been limited due to concerns about adverse events associated with long-term use of non-steroidal anti-inflammatory drugs. Several guidelines recommend moisturizing the skin and avoiding localized pressure as a preventive measure for capecitabine-induced HFS [15–17]. In clinical practice, treatment adjustments such as dose reduction and/or interruption are frequently required after the occurrence of HFS [18].

HFS development is considered to involve several mechanisms, including inhibition of skin basal cell proliferation; secretion of drugs through eccrine sweat glands; action of drug degradation products [9]; and inflammation mediated by interleukin (IL)-1a, IL-1b, IL-6, and reactive oxygen species [19]. Corticosteroids mitigate inflammation by suppressing the release of chemical mediators. A prospective study evaluating oral dexamethasone (8 mg/day with gradual tapering) for patients with pegylated liposomal doxorubicin-induced palmar-plantar erythrodysesthesia found that more patients receiving dexamethasone could continue therapy without dose modification than those not receiving dexamethasone [20]. In some case series, corticosteroids have been reported to be effective for cytarabine- or vinorelbine-induced HFS [21, 22]. Similarly, while skin rash associated with epidermal growth factor receptor inhibitors is partly attributed to inflammatory reactions [23–25], topical corticosteroids have been shown to prevent skin rash [26, 27]. In addition, a preventive effect of topical diclofenac gel on capecitabine-induced HFS has been reported [28]. Diclofenac, a non-steroidal anti-inflammatory drug, exerts anti-inflammatory effects similar to corticosteroids, which may contribute to the prevention of HFS.

Therefore, topical corticosteroids may prevent capecitabine-induced HFS. This study aimed to assess the preventive efficacy of medium-class topical corticosteroids (0.1% hydrocortisone butyrate) for capecitabine-induced HFS in patients receiving adjuvant chemotherapy for colorectal cancer.

## Methods

### Study design

This was an open-label, single-arm, single-center phase II study approved by the Clinical Research Review Board of the University of Tokyo (approval number: 2021512SP), conducted in compliance with the Clinical Trials Act of Japan and the declaration of Helsinki, and registered in the Japan Registry of Clinical Trials (jRCT) (registration number jRCTs031220002). The details of this study protocol have been reported elsewhere [29].

### Patients

The inclusion criteria were (i) curatively resected and histologically confirmed colorectal adenocarcinoma, (ii) pathological stage II or III disease, (iii) scheduled to receive adjuvant chemotherapy with CapeOX, (iv) age ≥ 18 years, (v) Eastern Cooperative Oncology Group (ECOG) performance status 0–1, (vi) no prior chemotherapy or radiotherapy, (vii) adequate organ function, and (viii) provision of written informed consent. In contrast, the exclusion criteria were (i) bacterial/fungal/spirochete/viral skin infections, (ii) eczema otitis externa with perforations in the eardrum, (iii) ulcers (excluding Behçet’s disease), second-degree or deeper burns/frostbite, (iv) other skin diseases, and (v) history of hypersensitivity to local hydrocortisone.

### Adjuvant chemotherapy

Adjuvant chemotherapy with CapeOX consisted of a 2-h intravenous infusion of oxaliplatin (130 mg/m^2^) on day 1, followed by oral capecitabine administration (1000 mg/m^2^, bid) from the evening of day 1 until the morning of day 15. This was repeated every 3 weeks up to four or eight cycles, depending on the physician’s discretion and/or patient’s preference. If necessary, dose interruption, schedule delay, dose reduction, and discontinuation were performed at the attending physician’s discretion. The capecitabine dose was reduced by 20% at one level; however, dose reduction by more than two levels was not allowed.

### Intervention

One fingertip unit (equivalent to 0.5 g) of 0.1% topical hydrocortisone butyrate and standard moisturizing therapy were applied to the palms and soles two times daily, once in the morning and evening, from day 1 and continued until the completion or discontinuation of adjuvant chemotherapy. All patients received education on standard self-care at the start of chemotherapy, which included keeping clean, moisturizing, wearing gloves, avoiding strenuous activity, preventing pressure, and avoiding direct sunlight.

### Criteria for discontinuing interventions

Interventions were discontinued in the following cases: (i) withdrawal of consent, (ii) capecitabine discontinuation, (iii) severe adverse events caused by 0.1% topical hydrocortisone butyrate, and (iv) administration of other prohibited therapy or drugs.

### Prohibited therapy and drugs

The following treatments and concomitant use of drugs were prohibited: (i) cancer treatment other than the CapeOX, (ii) other topical corticosteroids for the palms or soles, (iii) celecoxib administration, and (iv) pyridoxine administration.

### Evaluation

Patient symptoms were examined, and laboratory tests were performed at least once every 3 weeks; the severity of HFS and other adverse events was assessed by the clinical pharmacists and attending physicians, based on the self-reported adverse events [29]. Adverse events were graded according to the National Cancer Institute Common Terminology Criteria for Adverse Events version 5.0. The amount of hydrocortisone butyrate used (0.1%) was regularly monitored by clinical pharmacists who provided education to patients to promote adherence to the treatment protocol repeatedly.

### Endpoints

The primary endpoint was the incidence of grade ≥ 2 HFS within four cycles of the adjuvant chemotherapy. In contrast, the secondary endpoints were (i) incidence of each HFS grade within four and eight cycles of chemotherapy; (ii) time to onset of each HFS grade; and (iv) incidence of dose reduction, schedule delay, and capecitabine discontinuation due to any adverse event.

### Statistical analysis

For the primary endpoint, the incidence of grade ≥ 2 HFS was evaluated in the eligible patients. While the threshold was set at 40% based on the incidence of grade ≥ 2 HFS in control groups from previous RCTs investigating preventive strategies for capecitabine-induced HFS [12, 13, 30], topical application of hydrocortisone butyrate 0.1% was expected to reduce its incidence by 15%, targeting 25%. Most previous studies have reported control group incidences of approximately 30%, typically observed after 6 to 8 treatment cycles. However, Japanese patients are considered more susceptible to developing HFS [31]. Furthermore, the incidence of HFS tends to plateau after approximately four cycles [4, 32]. The minimum number of patients required to preserve a power of 70% was 39 (one-sided α = 0.1), and the planned sample size was 50 patients. Statistical analysis was performed using SAS Studio 3.8.

## Results

### Patient demographics

**Fig. 1:**
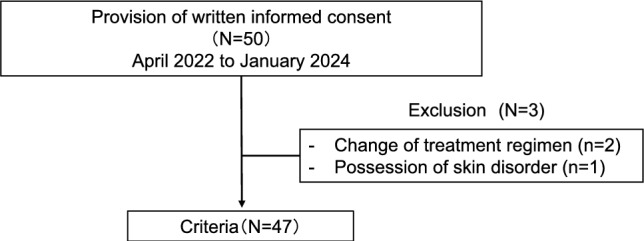
CONSORT diagram. Of the 50 patients enrolled, 47 met the eligibility criteria. Three patients were excluded: two receiving other chemotherapy and one with a skin disorder (detected after obtaining informed consent)

Fifty patients were enrolled between May 2022 and January 2024. Among these, two whose chemotherapy regimens were changed from CapeOx, and one with a skin disorder diagnosed between the time of obtaining informed consent and the start of the intervention, were excluded. Ultimately, 47 patients were included in the primary analysis (Fig. 1). The demographics of the included patients (n = 47) were as follows: median age of 54.5 (range, 28–79) years, 100% had Eastern Cooperative Oncology Group performance status 0, 36.1% were male, 95.7% had pathological stage III disease, and 40.4% had primary tumors located in the colon (Table 1). No patients experienced recurrence during the adjuvant chemotherapy.

**Table 1:** Demographics of the enrolled patients

Median age (range), years	54.5 (28–79)
Sex, n (%)	
Male	17 (36.1)
Female	30 (63.8)
ECOG performance status, no. (%)	
0	47 (100.0)
1	0 (0.0)
2	0 (0.0)
Primary tumor, no. (%)	
Cecum	1 (2.1)
Ascending colon	3 (6.4)
Descending colon	3 (6.4)
Sigmoid colon	9 (19.1)
Rectum	28 (59.6)
Pathological stage, no. (%)	
II	2 (4.3)
III	45 (95.7)

*ECOG* Eastern Cooperative Oncology Group

### Administration of chemotherapy

Overall, 43 (91.5%) completed four cycles of adjuvant chemotherapy, while 34 (72.3%) of the 47 completed eight cycles. The median relative dose intensity of capecitabine and oxaliplatin was 100 and 79.5% within four cycles, and 98.2 and 51.8% within eight cycles, respectively. During eight cycles of adjuvant chemotherapy, three patients (a total of 13 cycles) required a schedule delay of capecitabine due to nausea (two patients, a total of seven cycles) and diarrhea (one patient, a total of five cycles) (double count of cases with multiple reasons). Thirteen patients (a total of 65 cycles) required a dose reduction of capecitabine due to diarrhea (seven patients, a total of 14 cycles) and nausea (three patients, a total of three cycles). None of the patients required capecitabine discontinuation. For oxaliplatin, 13 patients (a total of 22 cycles) required a schedule delay of oxaliplatin due to neutropenia (eight patients, a total of 20 cycles), thrombocytopenia (eight patients, a total of 21 cycles), and peripheral neuropathy (seven patients, total nine cycles) (double count of cases with multiple reasons). Five patients (a total of 13 cycles) had a dose reduction of oxaliplatin due to nausea (three patients, a total of five cycles) and fatigue (three patients, a total of three cycles) (double count of cases with multiple reasons). One patient (a total of one cycle) skipped oxaliplatin once due to peripheral neuropathy, and 12 (a total of 45 cycles) discontinued oxaliplatin. The major reasons for discontinuation were peripheral neuropathy (six patients, total 28 cycles), thrombocytopenia (six patients, a total of 20 cycles), neutropenia (five patients, a total of 20 cycles), and anorexia (five patients, a total of 18 cycles) (double count of cases with multiple reasons). No adverse events induced by 0.1% topical hydrocortisone butyrate were observed (Table 2).

**Table 2:** Dose intensity of chemotherapy and dose modification

*Capecitabine*		
Median RDI^a^ % (range)		
Four cycles	100 (25.0–100)	
Eight cycles	98.2 (12.5–100)	
Schedule delay number		
Four cycles	1 (2.1)	
Eight cycles	3 (6.4)	
Dose reduction, number of patients (%)		
Four cycles	13 (27.7)	
Eight cycles	13 (27.7)	
Discontinuation, number of patients (%)		
Four cycles	0 (0.0)	
Eight cycles	0 (0.0)	
*Oxaliplatin*		
Median RDI% (range)		
Four cycles	79.5 (25.0–100)	
Eight cycles	51.8 (12.5–100)	
Schedule delay, number of patients (%)		
Four cycles	13 (27.7)	
Eight cycles	13 (27.7)	
Dose reduction, number of patients (%)		
Four cycles	5 (10.6)	
Eight cycles	5 (10.6)	
Discontinuation, number of patients (%)		
Four cycles	4 (8.5)	
Eight cycles	13 (27.7)	
Reasons for schedule modification of chemotherapy^b^, number of reasons		
	Capecitabine	Oxaliplatin
Schedule delay or lack of adherence		
Neutropenia	0	8
Thrombocytopenia	0	8
Peripheral neuropathy	1	7
Nausea	7	3
Liver function impairment	3	0
Hand-foot syndrome	2	0
Diarrhea	5	0
Pancreatitis	1	0
Fever	1	1
Anorexia	1	1
Taste disorder	1	0
Fatigue	2	0
Forgetting to take the agent	3	0
Dose reduction		
Hand-foot syndrome	0	0
Diarrhea	7	0
Nausea	3	3
Fatigue	1	3
Peripheral neuropathy	0	1
Anorexia	1	0
Neutropenia	0	1
Liver function impairment	1	0
Hand-foot syndrome	0	0
Interruption of administration		
Hand-foot syndrome	0	0
Peripheral neuropathy	0	6
Thrombocytopenia	0	6
Neutropenia	0	5
Anorexia	0	5
Nausea	0	4

In addition to the above, one case each of ileus and postponement due to the patient’s convenience was included

*RDI* relative dose intensity

^a^Adherence ratio of capecitabine was considered

^b^Double count in case of multiple reasons

### HFS

**Fig. 2:**
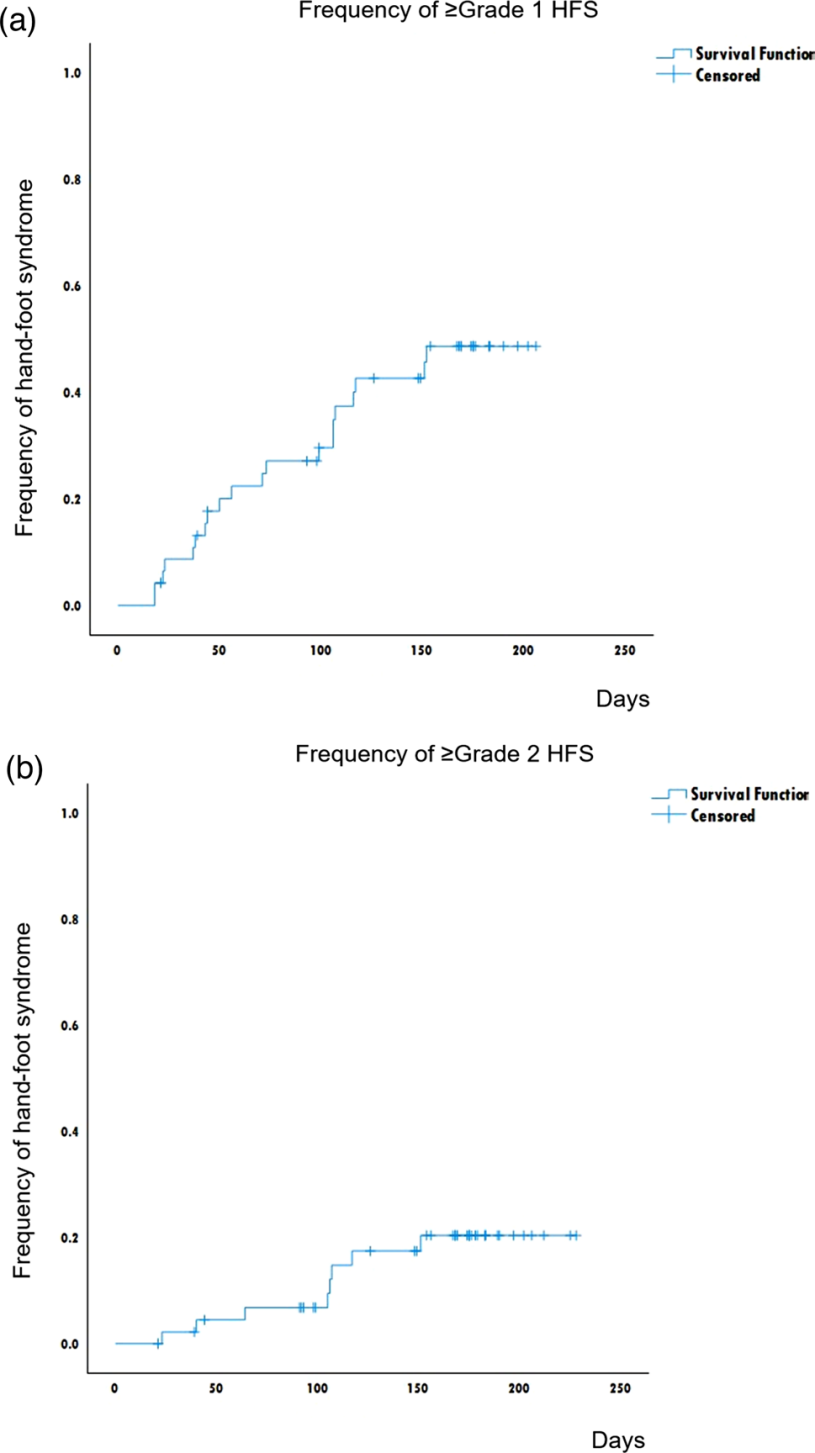
**a** The cumulative incidence of any grade of hand-foot syndrome (HFS) within 200 days. The incidence of any grade HFS within 200 days was 48.7% (23.7–64.4%). Median onset of HFS was 63.5 (18–152) days. **b** The cumulative incidence of grade ≥ 2 HFS within 200 days. The incidence of any grade HFS within 200 days was 20.45 (7.7–33.7%). Median onset of HFS was 105.5 (23–151) days. One patient had grade 3 HFS on day 164

Table 3 presents the incidence of each HFS grade within four and eight cycles. The incidence of grade ≥ 2 HFS within four and eight cycles was 6.4% (95% confidence interval [CI], 1.0–18.0%) and 17.0% (95% CI 8–31), respectively. Those of any grade HFS in four and eight cycles were 25.5% (95% CI 14.0–40.0) and 42.6% (95% CI 28.0–58.0), respectively. The times to onset of any grade HFS and grade ≥ 2 HFS were 63.5 (range 18–152) and 105.5 (range 23–151) days, respectively (Fig. 2a, b).

**Table 3:** Incidence of HFS

	n	%	95% CI
Four cycles			
Grade ≥ 1	12	25.5	14.0–40.0
Grade ≥ 2	3	6.4	1.0–18.0
Grade ≥ 3	0	0.0	0.0–8.0
Eight cycles			
Grade ≥ 1	20	42.6	28.0–58.0
Grade ≥ 2	8	17.0	8.0–31.0
Grade ≥ 3	1	2.1	0.0–11.0

*HFS* hand-foot syndrome, *CI* confidence interval, *NA* not available

### Adverse events

Table 4 presents the adverse events other than HFS. Adverse event related to 0.1% topical hydrocortisone butyrate was not observed. Peripheral sensory neuropathy (57.4%), neutropenia (46.9%), nausea (36.1%), anorexia (36.1%), and diarrhea (31.9%) were observed as major grade ≥ 2 non-hematological toxicities.

**Table 4:** Adverse events

	Any grade	Grade 1	Grade 2	Grade 3
	Number (%)			
Nonhematological toxicity				
Hand-foot syndrome	16 (34.0)	9 (19.1)	6 (12.8)	1 (2.1)
Peripheral sensory neuropathy	39 (83.0)	12 (25.5)	12 (25.5)	15 (31.9)
Diarrhea	25 (53.2)	10 (21.3)	5 (10.6)	10 (21.3)
Nausea	35 (74.5)	18 (38.3)	8 (17.0)	9 (19.1)
Vomiting	2 (4.3)	1 (2.1)	1 (2.1)	0 (0.0)
Anorexia	29 (61.7)	12 (25.5)	9 (19.1)	8 (17.0)
Taste disorder	11 (23.4)	10 (21.3)	1 (2.1)	0 (0.0)
Mucositis	12 (25.5)	10 (21.3)	1 (2.1)	1 (2.1)
Fatigue	38 (80.9)	31 (66.0)	4 (8.5)	3 (6.4)
Constipation	9 (19.1)	7 (15.0)	2 (4.3)	0 (0.0)
Pigmentation	14 (29.8)	14 (29.8)	0 (0.0)	0 (0.0)
Alopecia	1 (2.1)	1 (2.1)	0 (0.0)	0 (0.0)
Hiccup	4 (8.5)	4 (8.5)	0 (0.0)	0 (0.0)
Phlebitis	10 (21.3)	8 (17.0)	2 (4.3)	0 (0.0)
Insomnia	1 (2.1)	1 (2.1)	0 (0.0)	0 (0.0)
Fever	2 (4.3)	2 (4.3)	0 (0.0)	0 (0.0)
Edema	2 (4.3)	2 (4.3)	0 (0.0)	0 (0.0)
Increased aspartate aminotransferase levels	37 (78.7)	30 (63.8)	4 (8.5)	3 (6.4)
Increased alanine aminotransferase levels	26 (55.3)	19 (40.4)	4 (8.5)	3 (6.4)
Hematological toxicity				
Neutropenia	24 (51.1)	2 (4.3)	15 (31.9)	7 (15.0)
Anemia	32 (68.1)	27 (57.4)	5 (10.6)	0 (0.0)
Thrombocytopenia	28 (59.6)	18 (38.3)	8 (17.0)	2 (4.3)

## Discussion

Our study demonstrated a low incidence of grade ≥ 2 HFS (6.4%), with a median RDI of capecitabine at 100% within four cycles of chemotherapy, indicating favorable preventive effect of 0.1% topical hydrocortisone butyrate on capecitabine-induced HFS and higher dose intensity of capecitabine.

This study investigated the preventive effects of 0.1% topical hydrocortisone butyrate on capecitabine-induced HFS in patients with colorectal cancer receiving adjuvant CapeOX. In this study, the primary endpoint was the incidence of grade ≥ 2 HFS within four cycles (approximately 3–4 months), while the evaluation period of preventive effect for HFS was set as 3–6 months in previous RCTs [12, 13]. A previous phase III study of celecoxib reported that the mean time to the occurrence of grade 1 or 2 HFS was 4.3 months [13]. The median cumulative dose of capecitabine associated with grade ≥ 1 HFS was reportedly 100,000 mg/m^2^ [4], which corresponded to 3–4 cycles of CapeOX. Furthermore, the frequency of HFS has been reported to be stable after four cycles [32] in patients with colorectal cancer. Adjuvant chemotherapy with CapeOX for four cycles is an acceptable option for low-risk stage III colorectal cancer [33]. Therefore, we set the evaluation term in the primary endpoint as the incidence of grade ≥ 2 HFS during four cycles of adjuvant chemotherapy.

The incidence of grade ≥ 2 HFS within four cycles was 6.4% (95% CI 1–18%), indicating that the primary endpoint was met. Although this study was not a comparative trial, grade ≥ 2 HFS incidence rate tended to be lower than that of the control groups in other clinical trials [12, 13, 30]. In India, the D-TORCH study [28], which evaluated the preventive effect of 1% topical diclofenac gel on capecitabine-induced HFS, revealed that the incidence of grade ≥ 2 HFS within 3 months of initiating capecitabine-containing chemotherapy was 3.8%. This trial showed an incidence of grade ≥ 2 HFS within 3 months similar to that found in our study. The consistency between these two trials suggests that inflammation suppression—whether by topical steroid or diclofenac—provides effective prevention of capecitabine-induced HFS.

Additionally, the incidence of grade ≥ 2 HFS within eight cycles was 17.0% (95% CI 8–31%) in this study, which appeared to be < 11–52% reported in previous studies [10, 12, 13]. Among these studies, oral celecoxib reduced the incidence of grade ≥ 2 HFS to 11.8–14.7% [12, 13]. This study showed a comparable efficacy of 0.1% topical hydrocortisone butyrate on capecitabine-induced HFS with that of celecoxib [12, 13]. Considering celecoxib-related cardiovascular and gastrointestinal side effects, 0.1% topical hydrocortisone butyrate may be a safer alternative medication to prevent capecitabine-induced HFS.

Previous studies showed that any HFS grade developed within 3 [1] and 4.3 [13] months. Regarding grade ≥ 2 HFS, most cases experienced the symptoms within four cycles of CapeOX therapy (approximately 3 months) [4, 33]. Topical hydrocortisone butyrate (0.1%) did not appear to prolong the time to HFS occurrence. In this study, the times to onset of any grade and grade ≥ 2 HFS were 63.3 and 105.5 days, respectively, which were comparable to those of previous studies [4, 13]. This suggests that some mechanism other than inflammation, which cannot be prevented by steroids, contributes to HFS development.

In this study, the median dose intensity of capecitabine during four and eight cycles of chemotherapy was 100 and 98.2% respectively. The IDEA trial showed dose intensity of capecitabine as 91.2 and 78.0% for 3 and 6 months, respectively [34]. Dose intensity in this study appeared higher than that of the IDEA trial, particularly over 6 months. For up to eight cycles of adjuvant chemotherapy, 23.9% and 17.0% of patients with colorectal cancer required dose reduction and capecitabine discontinuation, respectively [31]. Similarly, large clinical trials reported capecitabine discontinuation in 15–30% of patients, with 5–12% due to HFS [1, 35]. In this study, while 13 (27.7%) patients required dose reduction of capecitabine mainly due to nausea and diarrhea, all participants continued capecitabine through eight cycles. No patients experienced recurrence during the adjuvant chemotherapy. These results suggest that primary prevention of HFS with topical steroids leads to completion of capecitabine, potentially improving treatment efficacy.

Concerns may arise regarding the prophylactic use of steroids. First, higher dose intensity could increase the incidence of capecitabine-induced adverse events other than HFS. However, in this study, the incidences of adverse events other than HFS were comparable to those reported in previous studies [2, 36]. Another concern is the potential for steroid-induced adverse events. However, no topical steroid-induced adverse event occurred in this study. The long-term application of oral steroids is discouraged for patients receiving chemotherapy since their continuous use sometimes induces infections. Topical steroids have a low risk of infection because they do not act systemically, and the safety of their long-term use is recognized [37, 38]. Furthermore, considering drug-drug interactions involving cytochrome P450 3A4 (CYP3A4), which is related to the metabolism of oral steroids, is not necessary. CYP3A4 inhibitors, such as aprepitant, azole, and clarithromycin, may lead to slowed metabolism and elevated blood concentration of oral steroids. Therefore, topical corticosteroids may have some advantages over oral corticosteroids.

This study has some limitations. First, as an open-label, single-arm study conducted at a single center, selection and evaluation biases cannot be ruled out. Second, the evaluation period for the primary analysis was as short as 3 months, limiting the assessment of long-term efficacy and adverse events of topical corticosteroids. Based on this study, a multi-center, double blind, RCT to verify the preventive effects of topical corticosteroids on capecitabine-induced HFS is under discussion.

## Conclusions

Topical hydrocortisone butyrate (0.1%) may present capecitabine-induced HFS with safe tolerability in capecitabine-containing chemotherapy.

## Data Availability

The data that support the findings of this study are available from the corresponding author, Yohei Iimura, upon reasonable request.

## Abbreviations

CapeOX: Capecitabine plus intravenous oxaliplatin
HFS: Hand-foot syndrome
jRCT: Japan Registry of Clinical Trials
RCT: Randomized controlled trial
